# Investigation of the relationship of general and digital health literacy with various health-related outcomes

**DOI:** 10.3389/fpubh.2023.1229734

**Published:** 2023-07-31

**Authors:** Éva Bíró, Ferenc Vincze, Gabriella Nagy-Pénzes, Róza Ádány

**Affiliations:** ^1^Department of Public Health and Epidemiology, Faculty of Medicine, University of Debrecen, Debrecen, Hungary; ^2^ELKH-DE Public Health Research Group, Department of Public Health and Epidemiology, Faculty of Medicine, University of Debrecen, Debrecen, Hungary; ^3^National Laboratory for Health Security, Center for Epidemiology and Surveillance, Semmelweis University, Budapest, Hungary; ^4^Department of Public Health, Semmelweis University, Budapest, Hungary

**Keywords:** general health literacy, digital health literacy, health behaviour, confidence in vaccination, self-perceived health, health care utilization, health literacy population survey 2019-2021

## Abstract

**Background:**

Despite the growing number of health literacy surveys, we know little about the combined effect of the different dimensions of health literacy on various health-related outcomes.

**Objective:**

Thus, our study aimed to examine the impacts of general and digital health literacy on health behaviour, confidence in vaccination, self-perceived health, and health care utilization.

**Methods:**

Our research was part of the Health Literacy Population Survey 2019–2021, which was an international, multicentre, cross-sectional study. The data were collected via computer-assisted telephone interview in December 2020 in Hungary. Multiple multinomial logistic and multivariate linear regression models were used to analyse the separately effects of general and digital health literacy on the studied outcomes. Moreover, the combined effect of general and digital health literacy was also analysed via sensitivity analyses. In the last step, the interactions between general and digital health literacy were examined using the Johnson-Neyman procedure.

**Results:**

The results did not reveal any associations between health literacy and health behaviour. Health care use was only affected by digital health literacy; however, this effect was inconsistent. Both dimensions of health literacy were positively associated with self-perceived health and vaccination confidence.

**Conclusion:**

Our results suggest that increasing health literacy could promote health and vaccination confidence, while the potential effect of higher digital health literacy on more conscious use of the health care system should be investigated further.

## 1. Introduction

Health literacy (HL) is a comprehensive concept (1) that – together with its public health importance – has been extensively discussed in the literature in the past decade. HL can be defined in many ways, here we are referring to the integrated model of HL (1) as the concept applied in the present study. According to the integrated model, HL “entails people’s knowledge, motivation and competences to access, understand, appraise, and apply health information in order to make judgments and take decisions in everyday life concerning healthcare, disease prevention and health promotion to maintain or improve quality of life during the life course” (1). In addition to the general term, an increasing number of special subtypes have emerged in recent years. In the current digital world, information and communication tools are available to almost everyone. The use of digital tools allows people to communicate, manage their tasks (e.g., make payments, use direct consumer services, access health services and medical records, book appointments) and access information quickly and easily. The internet also makes it easy to gain more knowledge about topics of interest, including health and its determinants, lifestyle changes, medicine or health care. However, the reliability and validity of the various types of information on the internet is often uncertain, especially since the emergence of social media platforms as major information-seeking and information-sharing channels for most individuals. Furthermore, it is challenging to identify accurate information. The COVID-19 pandemic highlighted that access to reliable information sources and services has become critical to enabling the general public to participate in health care and preventive decisions. Therefore, in addition to general health literacy (GHL), it is also essential to address electronic (eHL) or digital health literacy (DHL). The term eHL covers a set of skills – as the ability to read, use computers, search for and understand health information, and put it into context – needed to effectively engage information technology for health (2), while DHL means the ability to search for, access, understand, evaluate, validate, and apply online health information (3).

Previous studies have found that a higher level of HL is positively associated with better health behaviour, such as healthier dietary habits and regular physical activity (4–8). Aaby et al. also found an association between a low level of HL and being underweight or obese (7). Svendsen et al. established a similar relationship regarding body mass index, but the results regarding alcohol consumption and smoking were inconsistent (8). A higher level of HL has also been shown to be related to better perceived health status, preventive health behaviours – such as getting vaccinated or participating in health screenings (9–12) – and more appropriate use of health services (9, 13). Similarly, a higher level of DHL has been found to be associated with better self-reported health status (14) and health behaviour (15, 16). Low DHL can also be a potential contributor to the spread of online misinformation and to its devastating effects, as in the case of COVID-19-related information, including vaccine hesitancy. Therefore, surveying and improving the level of DHL could be a potential tool for future pandemic and infodemic preparedness (17).

Due to the importance of HL, in 2018, the World Health Organization (WHO) Regional Office for Europe established the Action Network on Measuring Population and Organizational Health Literacy (M-POHL), which aims to support internationally comparable data collections on HL and to use the results for recommendations regarding interventions and actions (3). The M-POHL’s first project was the Health Literacy Population Survey Project 2019–2021 (HLS_19_), which measured the HL of the population in 17 countries (including Hungary) of the WHO European Region (3). The HLS_19_ assessed not only GHL but also DHL, and this survey found that GHL is related to DHL, and DHL is correlated with health status and use of health services (3).

To our knowledge, previous studies have not performed a detailed investigation of the relationship between GHL and DHL or their combined impact on health-related outcomes. Our study aimed to examine the effects of DHL and GHL on health behaviour, confidence in vaccination, self-perceived health, and health care utilization based on data collected in accordance with an international survey protocol.

## 2. Materials and methods

### 2.1. Study design and sampling

Our survey was part of the Health Literacy Population Survey 2019–2021 (HLS_19_), an international multicentred cross-sectional study. The investigation was conducted in line with the international protocol in December 2020 in Hungary. The data used in the present article were derived from a probability sample that had been selected in multiple stages with proportional stratification. As a first step in sampling allocation, the regions were selected as the first strata, followed by the type of settlements as the second. Then, the localities were chosen from these strata with the help of probability-proportional-to-size sampling; afterwards, the number of respondents who should get into the sample from each type of settlement within each region was determined. In the last step, when the sample was complied, a two-level strata-design was used where the region/settlement type was the first level and the respondents’ gender/age-group/educational level/settlement type was the second level, resulting in a probability sample that was representative of the Hungarian population aged 18 years and older who are living in private households stratified by age, sex, education, and settlement type. To collect information, questionnaires were administered by trained interviewers via computer-assisted telephone interview (CATI) due to the COVID-19 pandemic. The study was approved by the Medical Research Council Scientific and Research Committee, Hungary (IV/10181–3/2020/EKU). Informed consent was obtained from all participants in accordance with the Declaration of Helsinki.

### 2.2. Studied variables

#### 2.2.1. Sociodemographic variables

Regarding the study aims, the following sociodemographic variables were used: age, sex, marital status, education level, employment status, and perceived socioeconomic status. The questions were the same as in previous national and international questionnaire-based surveys (18–20). The following age groups were examined: 18–24, 25–34, 35–44, 45–54, and 55–64. Marital status was dichotomized as married and other (single or divorced or separated or widowed). The highest level of education was categorized as primary or less, lower secondary, secondary, bachelor or tertiary, and master or doctoral school, while the employment status was described with the following categories: employed, student, unemployed and other (retired, unable to work or domestic). Socioeconomic status was measured with the self-perceived level in society (a 10-point Likert scale with answers from “1” – lowest level to “10” – highest level in society). In the descriptive analyses, the scale was categorized as follows: not well off (1–4 points), average (5 points), and well off (6–10 points). During the explanatory analyses, socioeconomic status was treated as a continuous variable.

#### 2.2.2. Health literacy

In the HLS_19_ survey to measure GHL, a short questionnaire, the HLS_19_-Q12 (21), was developed to collect data. This questionnaire measures people’s perceptions of their ability to obtain, understand, evaluate, and use health information to preserve and enhance their health with 12 questions. The answers were measured on a four-point Likert scale from “very easy” to “very difficult.”

In the HLS_19_ survey, DHL covers the “ability to search for, access, understand, appraise, validate, and apply online health information, and the ability to formulate and express questions, opinion, thoughts, or feelings when using digital devices” (22). It is measured by eight items to be rated on a four-point Likert scale ranging from “very easy” to “very difficult.”

Regarding the international protocol (3), the final GHL and DHL score is calculated as the percentage (0–100) of valid items with responses that were answered with “very easy” or “easy.” If more than 20% of the items contained invalid responses, the score was set to “missing.” A higher score indicates a higher level of DHL and GHL. Furthermore, in this studied sample, both GHL and DHL had high internal reliability (Cronbach’s α_GHL_ = 0.823, Cronbach’s α_DHL_: 0.820) and high split-half reliability (Spearman-Brown coefficient_GHL_ = 0.836, Spearman-Brown coefficient_DHL_ = 0.864).

### 2.3. The studied outcomes

#### 2.3.1. Health behaviour

Health behaviour was evaluated through four variables: smoking habits, alcohol consumption, physical activity and fruit or vegetable consumption. The questions were the same as in the European Health Interview Survey (18, 19). All questions were measured on an eight-item scale from “never” to “7 days a week.” The frequency of use of tobacco and alcohol use was assessed with two questions (during a typical week how many days do you use tobacco product/drink alcohol). For tobacco use, the answers were classified into three categories: nonsmoker, not a daily, daily smoker. For alcohol use, the answers were also classified into three categories: abstinent, not a weekly consumer, consume at least weekly. Respondents were asked about healthy food consumption – specifically, they were asked how many days a week they eat fruit or vegetables. Answers were classified as 7 days per week, 3–6 days per week and less than 3 days per week. The regularity of physical activity was measured using a question regarding the number of days when the respondent was physically active for at least 30 min that led to an increase in breathing or heart rate. The answers were categorized as follows: maximum 6 days per week, 7 days per week and fully inactive.

#### 2.3.2. Health care use, confidence in vaccination and self-perceived health

The variables related to the respondent’s health care use included the number of episodes of emergency service use, the number of doctor (general practitioner, GP) visits, the number of medical specialist visits, the use of inpatient services (the number of admissions to inpatient care) and the number of admissions to day-patient services. All the health care utilization questions referred to the past 12 months except the one about the emergency service, where the time period was 24 months. These questions were all taken from the Hungarian implementation of the 2019 European Health Interview Survey, and health care utilization was treated as a categorical variable with cut-off values determined by the 25th, 50th, and 75th percentiles of the answers (18, 19).

“Confidence in vaccination” was assessed by using the shortened 4-item version of the Vaccine Confidence Index (23, 24), which measures confidence in vaccinations in terms of their importance, safety, and effectiveness. The items ask respondents to rate the extent to which they agree (“strongly agree” to “strongly disagree”) that vaccinations are important to protect themselves and their children and the extent to which they agree that vaccinations are safe and effective. This block was supplemented in the HLS_19_ survey by an additional item on the importance of vaccinations (to prevent the spread of diseases). We calculated the mean of the individual items’ scores and treated the confidence in vaccination as a continuous variable. The reliability analyses showed good consistency (Cronbach’s α: 0.903, Spearman-Brown coefficient = 0.889). Furthermore, participants were asked to rate their health status (“How do you rate your health status overall?) on a scale from 1 (very bad) to 5 (very good), and this information was used as a continuous scale (18, 19).

### 2.4. Statistical analyses

Descriptive statistics were used to describe the unweighted sample characteristics. Due to the complex sampling design, the weighted means (with the corresponding 95% confidence intervals (95% CI)) of DHL and GHL were reported and were stratified by the major sociodemographic categories. Then, we used multiple multinomial logistic and multivariate linear regression models to separately analyse the effects of DHL and GHL on the studied health behaviour, health care use, vaccination confidence and self-perceived health outcomes. Moreover, in sensitivity analyses, the combined effect of the two dimensions of HL was also analysed as a main effect (sensitivity analysis I) and as an interaction effect (sensitivity analysis II). All the studied model estimates were controlled for the respondents’ sociodemographic characteristics. Associations were quantified by regression coefficients (β) and odds ratios (OR).

In the last step, the significant interactions were probed using the Johnson-Neyman procedure with 95% confidence intervals. The Johnson-Neyman plot specifies the range of values of the moderator in which the slope of the predictor is significant vs. nonsignificant at a specified alpha level (25). The procedure interpreted to determine the levels of GHL at which DHL slopes were significant. The interactions were examined using R 4.0.5 (26), the stats, and interactions packages (27); the other analyses were carried out using SPSS 26.0 (IBM Corp, Armonk, NY, United States) software.

## 3. Results

### 3.1. Descriptive characteristics of the sample

A total of 1,195 subjects participated in the Hungarian HLS_19_ survey in 2020. The use of information and communication technology in the older adult population is significantly less frequent than that in the general population, so the sample was restricted to 18-to 64-year-old respondents, which resulted in a sample of 845 persons. The statistical analysis was restricted to participants who provided complete information on all sociodemographic variables. There were fifteen participants with missing sociodemographic data who were excluded from the analysis; ultimately, the analyses included 830 subjects.

A total of 28.55% of patients were aged 55–64 years, and 51.81% of patients were female. The majority of the respondents had completed secondary education (36.51%) and were married (66.14%). Regarding socioeconomic status, approximately 26% of the sample belonged to the not well-off category, and the unemployment rate was 4.94% (Table 1). Table 1 also shows the health literacy characteristics of the participants stratified by major sociodemographic characteristics. Overall, the GHL score (79.46 (95% CI = 78.22–80.70)) was significantly higher than the DHL score (72.89 (95% CI = 71.14–74.64)). Among the 18–24 age group, the GHL level was significantly lower compared to the older (45–54 and 55–64) age groups, while only a small, nonsignificant difference was observed between the sexes. The weighted average of GHL was significantly higher among those with a bachelor’s or tertiary level education than among those with only a primary level of education. Socioeconomic status had a positive effect on both DHL and GHL, and there were significant differences between individuals in the lower and higher socioeconomic strata.

**Table 1:** Sociodemographic characteristics of respondents and the weighted differences in health literacy stratified by major sociodemographic characteristics.

		Total number of valid responses (%)*	Weighted responses %	Digital health literacy score**	General health literacy score**
Age group (years)	18–24	67 (8.07%)	11.05%	67.95 (62.30–73.61)	73.17 (69.05–77.30)
	25–34	147 (17.71%)	24.02%	71.99 (68.25–75.73)	78.26 (75.89–80.62)
	35–44	164 (19.76%)	22.08%	73.43 (69.93–76.92)	79.99 (77.14–82.83)
	45–54	215 (25.90%)	22.09%	76.72 (73.16–80.27)	82.02 (79.47–84.57)
	55–64	237 (28.55%)	20.76%	72.01 (68.02–76.00)	80.91 (78.36–83.46)
Sex	Male	400 (48.19%)	48.84%	71.83 (69.31–74.34)	80.21 (78.56–81.86)
	Female	430 (51.81%)	51.16%	73.87 (71.42–76.32)	78.75 (76.91–80.58)
Education level	Maximum primary education	97 (11.69%)	14.37%	68.86 (63.53–74.19)	75.91 (72.04–79.78)
	Lower secondary education	229 (27.59%)	33.32%	70.89 (67.78–73.99)	78.18 (75.86–80.49)
	Secondary education	303 (36.51%)	33.43%	75.70 (72.85–78.55)	80.95 (78.95–82.95)
	Bachelor or tertiary education	103 (12.41%)	8.93%	74.12 (68.86–79.38)	83.02 (79.96–86.08)
	Master’s or doctoral or equivalent level	98 (11.81%)	9.95%	73.92 (68.27–79.56)	80.68 (77.43–83.93)
Marital status	Married	549 (66.14%)	63.39%	73.74 (71.64–75.85)	80.41 (78.94–81.89)
	Single/divorced/separated/widowed	281 (33.86%)	36.61%	71.37 (68.25–74.49)	77.81 (75.60–80.02)
Socioeconomic status	Not well-off	213 (25.66%)	27.20%	67.34 (63.67–71.02)	75.58 (72.95–78.21)
	Average	282 (33.98%)	33.79%	73.31 (70.26–76.37)	79.91 (77.84–81.98)
	Well-off	335 (40.36%)	39.01%	76.08 (73.51–78.65)	81.77 (79.93–83.61)
Employment status	Employed	632 (76.14%)	76.10%	73.41 (71.47–75.35)	79.95 (78.54–81.36)
	Student	35 (4.22%)	5.73%	67.22 (58.80–75.64)	70.31 (64.86–75.75)
	Retired, unable to work or domestic	122 (14.70%)	12.15%	74.42 (68.73–80.12)	80.69 (76.90–84.49)
	Unemployed	41 (4.94%)	6.01%	68.65 (60.61–76.69)	79.44 (75.38–83.50)
TOTAL		830 (100.00%)	–	72.89 (71.14–74.64)	79.46 (78.22–80.70)

*Valid case numbers (unweighted percentages), **Weighted means (weighted 95% confidence intervals) based on population estimates.

### 3.2. Association of health literacy with health behaviour, health status and health care utilization

The results of the multivariate multinomial logistic regression indicate that individuals with higher DHL had significantly greater odds of eating fruit and vegetables 7 days per week (OR = 1.008; *p* = 0.045) or 3–6 days per week (OR = 1.009; *p* = 0.040) than individuals with lower DHL. The odds of a lower number of medical specialist visits (OR = 1.009; *p* = 0.046) were higher among respondents with greater DHL. Furthermore, greater GHL increased the odds of eating fruits and vegetables 7 days per week (OR = 1.013; *p* = 0.019). When GHL and DHL were analysed in the same model, we found that higher DHL increased the odds of fewer medical specialist visits (OR = 1.010; *p* = 0.047) and fewer GP visits (OR = 1.008; *p* = 0.047) and decreased the chance of less frequent emergency care service use (OR = 0.984; *p* = 0.031), independent of GHL and the respondents’ sociodemographic factors. In the combined models, GHL had no effect on the studied variables, and the interaction between DHL and GHL was also nonsignificant (Table 2).

**Table 2:** The link between digital and general health literacy scores and the different health behaviour and health care use variables, estimated with multivariate multinomial logistic regression models, controlling for the sociodemographic status of the respondents.

		Digital health literacy			General health literacy			Digital health literacy (sensitivity analysis I)			General health literacy (sensitivity analysis I)		
		OR	SE	*p* value	OR	SE	*p* value	OR	SE	*p* value	OR	SE	*p* value
Smoking status ref.: daily smoker	Not daily smoker	1.003	0.006	0.595	0.994	0.008	0.431	1.006	0.007	0.398	0.992	0.009	0.391
	Nonsmoker	1.002	0.003	0.559	1.004	0.004	0.399	1.000	0.004	0.958	1.005	0.005	0.342
Alcohol consumption ref.: weekly user	Not weekly use	0.999	0.004	0.762	1.009	0.005	0.101	0.995	0.005	0.246	1.012	0.007	0.069
	Abstinent	1.005	0.004	0.254	1.009	0.005	0.097	1.001	0.005	0.786	1.010	0.007	0.146
Physical activity ref.: full inactive	Maximum six days per week	0.997	0.005	0.543	1.000	0.006	0.964	0.996	0.006	0.483	1.003	0.008	0.724
	Seven days per week	1.004	0.005	0.457	1.003	0.006	0.652	1.003	0.006	0.608	1.003	0.008	0.750
Fruit and vegetable consumption ref.: two days per week or less	Three to six days per week	**1.009**	**0.004**	**0.040**	1.007	0.006	0.194	1.008	0.005	0.131	1.003	0.007	0.635
	Seven days per week	**1.008**	**0.004**	**0.045**	**1.013**	**0.005**	**0.019**	1.005	0.005	0.345	1.011	0.007	0.110
Emergency services use ref.: two or more	Not used	0.992	0.006	0.154	1.007	0.007	0.325	0.987	0.007	0.052	1.012	0.008	0.127
	One times	0.989	0.007	0.085	1.002	0.008	0.787	**0.984**	**0.008**	**0.031**	1.014	0.010	0.168
General practitioner visits ref.: three or more	Not used	1.003	0.004	0.481	1.000	0.005	0.957	1.004	0.004	0.341	0.996	0.006	0.481
	One or two times	1.006	0.004	0.088	1.000	0.005	0.918	**1.008**	**0.004**	**0.047**	0.994	0.006	0.297
Medical specialist visits ref.: two or more	Not used	1.001	0.003	0.759	1.005	0.004	0.234	1.000	0.004	0.976	1.003	0.005	0.544
	One times	**1.009**	**0.004**	**0.046**	1.006	0.006	0.285	**1.010**	**0.005**	**0.047**	0.997	0.007	0.639
Inpatient services use ref.: two or more	Not used	0.995	0.006	0.382	1.003	0.008	0.670	0.991	0.007	0.185	1.011	0.009	0.214
	One times	1.028	0.019	0.145	1.005	0.020	0.805	1.034	0.021	0.117	0.986	0.023	0.541
Day-patient services use ref.: two or more	Not used	1.000	0.009	0.966	1.011	0.012	0.390	0.996	0.010	0.677	1.015	0.014	0.291
	One times	0.997	0.011	0.773	1.004	0.014	0.769	0.993	0.012	0.588	1.011	0.017	0.497

ref., reference category; sensitivity analysis I, both digital and general health literacy included in the model as a main effect; OR, odds ratios, controlled for age, sex, education, socioeconomic status, employment and marital status; SE, standard error; significant results marked in bold; all of the studied interactions between digital and general health literacy were nonsignificant; therefore, the results were excluded from the table.

According to the linear regression models, DHL was positively associated with self-perceived health (*β* = 0.001; *p* = 0.031) and vaccination attitude (*β* = 0.003; *p* < 0.001), and there was a positive link between GHL and self-perceived health (*β* = 0.003; *p* = 0.001) and vaccination attitude (*β* = 0.004; *p* < 0.001). When both HL scales were included in one model, greater DHL was associated with greater vaccination attitude (*β* = 0.003; *p* = 0.005), and increased GHL was associated with increased self-perceived health (*β* = 0.003; *p* = 0.011) and vaccination attitude (*β* = 0.003; *p* = 0.046). Finally, the interaction between GHL and DHL was associated with increased levels of self-perceived health (*β* = 1.77E-05; *p* < 0.004) and vaccination attitude (*β* = 3.66E-05; *p* < 0.001) (Table 3).

**Table 3:** The link between digital and general health literacy scores and the self-perceived health and vaccination attitude, estimated with multivariate linear regression models, controlling for the sociodemographic status of the respondents.

	Digital health literacy			General health literacy			Digital health literacy (sensitivity analysis I)			General health literacy (sensitivity analysis I)			Interaction of health literacy scores (sensitivity analysis II)		
	*β*	SE	*p* value	*β*	SE	*p* value	*β*	SE	*p* value	*β*	SE	*p* value	*β*	SE	*p* value
Self-perceived health	**0.001**	**0.001**	**0.031**	**0.003**	**0.001**	**0.001**	0.001	0.001	0.499	**0.003**	**0.001**	**0.011**	**1.77E-05**	**6.08E-06**	**<0.004**
Vaccination confidence	**0.003**	**0.001**	**<0.001**	**0.004**	**0.001**	**<0.001**	**0.003**	**0.001**	**0.005**	**0.003**	**0.001**	**0.046**	**3.66E-05**	**7.40E-06**	**<0.001**

Sensitivity analysis I, both digital and general health literacy included in the model as a main effect; sensitivity analysis II, both digital and general health literacy included in the model as an interaction effect; *β*, regression coefficients, controlled for sex, age, education, socioeconomic status, employment and marital status; SE, standard error; significant results marked bold.

**Figure 1:**
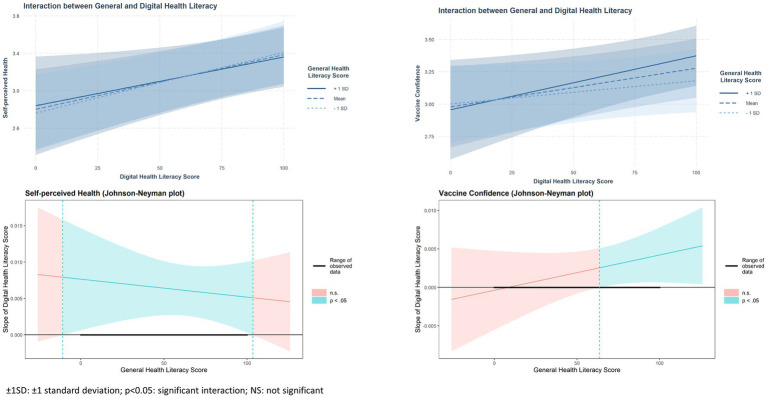
Interaction effect of general and digital health literacy on self-perceived health and vaccination attitude estimated with interaction and Johnson-Neyman plots with 95% confidence bands.

The plots in Figure 1 demonstrate the interaction of self-perceived health and vaccination attitude with DHL at 1 standard deviation above and below the average GHL. The results indicated that as the GHL increased, the slope of the DHL level also increased. The Johnson-Neyman technique suggested that when the GHL exceeded 63.58 points, the DHL level had a significant positive adjusted effect on vaccination confidence. Additionally, over the entire range of GHL scores, the DHL level had a significant increased adjusted effect on self-perceived health.

## 4. Discussion

In this study, we examined the effects of GHL and DHL on health behaviour, health care utilization, confidence in vaccination, and self-perceived health outcomes in different models by analysing the separate effects of DHL and GHL and their combined effect and by taking into consideration the potential interaction between GHL and DHL.

We did not find any associations of smoking and alcohol consumption with HL in any of the models examined. These results may be explained by the fact that HL is not a strong determinant of addictive behaviours, or in other words, the addictive effect of these substances has more influence on lifestyle factors than the level of health literacy. According to a recent meta-analysis, the odds of smoking are higher among those with inadequate HL, but this association was also dependent on the HL questionnaire used (28); therefore, the results are not consistent. There was no association between physical activity and GHL or DHL. Our results indicate that individuals with higher DHL have significantly greater odds of eating fruits and vegetables more frequently than individuals with lower DHL, and greater GHL increased the odds of eating fruits and vegetables every day. However, this association disappeared when both subtypes of HL were integrated into one model. Previous studies have also found a correlation between higher DHL and healthier diets (such as more fruit, vegetables, and nutrient intake) as well as balanced diets (16, 29, 30). A recent meta-analysis found that the correlation between DHL and health behaviours was weak in the age group examined in the current study, but the correlation was stronger in the above 65 age group (15). This finding may explain no association between behaviour and HL was observed in the current study; additionally, the measurement tool used herein to examine these behaviours was not able to provide detailed information. These could be the reasons that the previously assumed associations between health behaviour and HL was not proved in our study.

Both GHL and DHL were found to be positively associated with self-perceived health and vaccination confidence, as expected. According to our combined model, greater DHL was associated with greater vaccination confidence, and increased GHL was associated with increased self-perceived health and increased vaccination confidence. Based on the results obtained in our analysis, DHL and GHL reinforce each other for self-perceived health: the interaction effect was strong when both GHL and DHL are low as well as when both GHL and DHL are high. This is not the case with vaccination confidence; for this outcome, mutually reinforcing effects were only observed if the respondent had a GHL value of at least 63.58. Otherwise, the relationship between DHL and GHL is not relevant. This means that it is important to increase both GHL and DHL to achieve better self-perceived health and vaccination confidence. Previous studies have also shown a relationship between HL and higher vaccination confidence (31) or lower vaccine hesitancy (32–34). We have not found any other article with the same method of data analysis, which is why we cannot compare our results directly with previous ones; however, other studies have reported a positive relationship between the level of HL and health status or getting vaccines (11, 12, 35, 36), so we can say that our results are in line with earlier studies.

We found a relationship between DHL and health care utilization: higher DHL increased the odds of a lower number of medical specialist and GP visits and decreased the chance of less frequent emergency care service use. When comparing these results with previous findings, the exact relationship between HL and the use of health services is not clear (37). One possible explanation can be that those with higher DHL have better knowledge about the signs that indicate a strong need for emergency care, and they are also informed about how to address certain conditions or prevent diseases, which is why they do not use primary and outpatient care so often. Moreover, we must also take into consideration that our sample consisted of relatively younger people, who probably have not had as much contact with the health care system. Based on the results of a Belgian study (38), where health care use was measured objectively, there was a connection between the level of GHL and the use of certain health care services, such as general hospitals, one-day clinics, GP home visits, psychiatrist consultations and ambulance transportation. That study found no associations between health literacy and other health care services, such as admissions to one-day surgical clinics, consultations with the GP, and emergency services. They argued that a potential reason for these differences could be whether the type of consultation requires a high level of health competence on the part of the patients or not. Other studies also found no association between GHL and the use of emergency services and hospitals (39, 40). These results might be explained by the characteristics of the health care system (such as affordability and accessibility) in each country, which can influence the frequency of using different health services and the comparability of surveys carried out in other countries.

### 4.1. Strengths and limitations

This investigation has some limitations. First, the cross-sectional nature of the study is not precise enough to draw conclusions about causal relationships, and the use of self-reported data (e.g., in the case of health care use) could also influence the potential findings. Furthermore, this investigation could not cover the population aged 65 and older; therefore, additional targeted investigations would be necessary to describe the older Hungarian population’s DHL. The comparability of the results with other studies could be limited by the different data collection tools, statistical analyses (crude or adjusted), and differences in the work and accessibility of the health care systems in different countries.

Among the strengths of the study, it should be mentioned that during the nationwide representative sampling and data collection, an international guideline was followed, which likely reduced the risk of systematic errors. Furthermore, to our knowledge, this is the first study in which the effects of GHL and DHL on different health outcomes were investigated together.

## 5. Conclusion

Our results revealed that independent of several sociodemographic factors, DHL and GHL strengthen each other in the case of self-perceived health, while in the case of vaccination confidence, we found a theoretical minimum GHL score (63.58), below which the reinforcing relationship between DHL and GHL is nonsignificant. It may be essential to consider this relationship for vaccination campaigns when preparing for potential future pandemics. To achieve higher confidence, novel techniques and tools should focus first on increasing GHL and then increasing DHL.

The results of our study suggest that increasing DHL and GHL could promote general health and vaccination confidence among those below the age of 65, while the potential effect of higher DHL on more conscious use of the health care system should be investigated further. The lack of HL is a public health challenge, and it is vital to take the necessary steps to increase HL at all levels (41) because it can be considered a modifiable risk factor for health disparities (42).

## Data availability statement

The raw data supporting the conclusions of this article will be made available by the authors, without undue reservation.

## Ethics statement

The studies involving human participants were reviewed and approved by Medical Research Council Scientific and Research Committee, Hungary. Written informed consent for participation was not required for this study in accordance with the national legislation and the institutional requirements.

## Author contributions

ÉB contributed to the conception of the work, supervision of data collection, interpretation of data, and drafting the article. FV contributed to the conception of the work, data analysis, interpretation of data, visualization, and drafting the article. GN-P contributed to the conception of the work, interpretation of data, and drafting the article. RÁ acquired funding and contributed to the conception of the work, supervision, and critical revision of the article. All authors contributed to the article and approved the submitted version.

## Funding

The work was supported by the Ministry of Human Capacities, Hungary (IV/956-4/2020/EKF). This paper was supported by the János Bolyai Research Scholarship of the Hungarian Academy of Sciences. This project was also financed by the Eötvös Loránd Research Network (TKCS-2021/32). Project no. 135784 has also been implemented with the support provided by the National Research, Development, and Innovation Fund of Hungary, financed under the K_20 funding scheme. RÁ also works as team member of the National Laboratory for Health Security Hungary (RRF-2.3.1-21-2022-00006) supported by the National Research, Development and Innovation Office (NKFIH).

## Conflict of interest

The authors declare that the research was conducted in the absence of any commercial or financial relationships that could be construed as a potential conflict of interest.

## Publisher’s note

All claims expressed in this article are solely those of the authors and do not necessarily represent those of their affiliated organizations, or those of the publisher, the editors and the reviewers. Any product that may be evaluated in this article, or claim that may be made by its manufacturer, is not guaranteed or endorsed by the publisher.
